# Spatial Tools for Integrated and Inclusive Landscape Governance: Toward a New Research Agenda

**DOI:** 10.1007/s00267-021-01547-x

**Published:** 2021-10-15

**Authors:** Mirjam A. F. Ros-Tonen, Louise Willemen, Michael K. McCall

**Affiliations:** 1 Department of Geography, Planning and International Development Studies, University of Amsterdam, Nieuwe Achtergracht 166, 1018 VW Amsterdam, The Netherlands; 2 Faculty of Geo-Information Science and Earth Observation (ITC), University of Twente, Hengelosestraat 99, 7514 AE Enschede, The Netherlands; 3 Centro de Investigaciones en Geografia Ambiental (CIGA), Universidad Nacional Autónoma de Mexico, Antigua Carretera a Pátzcuaro 8701, 58190 Morelia, Mich Mexico

**Keywords:** Participatory spatial tools, Integrated landscape approaches, Inclusive landscape governance.

## Abstract

Participatory spatial tools—community mapping, PGIS, and others—find increasing resonance among research and non-governmental organizations to make stakeholder claims and community perspectives explicit for more inclusive landscape governance. In this paper, we situate the use of participatory spatial tools in debates on integrated landscape approaches and inclusive development. We show that using such spatial tools is not new but argue that their application for inclusive landscape governance requires a new research agenda that focuses on expanding the scope of application of the tools, improving the inclusivity of the processes, and developing new technologies.

## Introduction

The growing demand for food and non-food crops and rural land for other uses is increasing the dynamics and complexity of landscapes, affecting rural and peri-urban land use, ecosystem services, and livelihoods in multiple interacting ways (Shackleton et al. 2019; Macchi et al. 2020). For example, expanding cocoa and oil palm cultivation in countries like Ghana or Indonesia alters forest cover and biodiversity and the associated availability of non-timber forest products that supplement people’s dietary diversity and livelihoods (Asubonteng et al. 2018; Santika et al. 2019). There is a continuing call in scientific and policy arenas to address the negative effects of landscape changes in a holistic and integrated manner (Sayer et al. 2013; Djenontin et al. 2021). Such integrated approaches are considered vital to achieving the Sustainable Development Goals (Reed et al. 2015; Thaxton et al. 2015; DeClerck et al. 2016).

Integrated landscape approaches look at the whole (rural) landscape to find solutions that meet manifold demands by taking into account the dynamics, synergies, and trade-offs among the multiple objectives of numerous stakeholders in specific land units (Sayer et al. 2013; Ros-Tonen et al. 2018; Reed et al. 2020a).^1^ While many terms exist for landscape approaches (Scherr et al. 2012; Reed et al. 2014), the common denominators are place-based and cross-sector stakeholder negotiation and engagement, multi-objective decision-making, and governance oriented toward achieving multifunctional landscapes. Such landscapes simultaneously provide food security, biodiversity and ecosystem services, sustainable livelihoods, and climate resilience (Sayer et al. 2013). A deep understanding of the landscape and recognizing the need for inclusivity, and hence strong stakeholder collaboration at different spatial levels, form the basis of this approach (Sayer et al. 2013; van Oosten et al. 2014; Reed et al. 2020b).

The call for integrated approaches is not new, as exemplified by the literature on integrated rural development (e.g., Ruttan 1984), integrated water(shed) management (e.g., Pahl-Wostl et al. 2008; Heathcote 2009), integrated natural resource management (e.g., Campbell and Sayer 2003), and integrated conservation and development projects (Brandon and Wells 1992; Alpert 1996). However, the new wave of integrated landscape approaches focuses more than previous approaches on making the trade-offs between land uses explicit and subject to negotiation among stakeholders. Acknowledging conservation-development trade-offs, the ‘new’ integrated landscape approaches aim to achieve ‘win more and lose less’ outcomes rather than elusive ‘win-wins’ (Reed et al. 2016; Ros-Tonen et al. 2018).

Negotiating trade-offs in an inclusive manner requires tools to unearth the views of the stakeholders. Therefore, participatory mapping and other spatial tools are increasingly applied to support place-based planning, including in urban contexts (e.g., Miranda Azeiteiro et al. 2017; Nadin et al. 2021; Pfeffer et al. 2013) and in water management (e.g., Van Cauwenbergh et al. 2018). They find resonance among international organizations and conservation NGOs for making landscape services and benefits and their associated claims and cultural values spatially explicit (e.g., Palomo et al. 2018; Fagerholm et al. 2019; Movik et al. 2021). An increasing body of literature (see also the papers in this special issue on Spatial tools for integrated and inclusive landscape governance^2^) proposes the use of participatory spatial tools such as participatory mapping, participatory geographical information systems (PGIS), and place-based scenario-building to uncover and visualize different stakeholder perspectives of landscape dynamics and associated consequences as a basis to negotiate solutions. Much of this literature can be positioned in the debate on integrated landscape approaches. However, a more critical scholarship warns that maps may be selective in their representations and obscure the complexities of land rights and power struggles over land use and resources (e.g., Sletto et al. 2020; Best et al. 2021; Movik et al. 2021; Windey and Van Hecken 2021). This paper, therefore, highlights some critical notes regarding the application of participatory spatial tools.

In the remainder of this paper, we first elaborate on the concept of integrated and inclusive landscape governance. Next, we highlight the drivers behind the growing use of participatory spatial tools in landscape governance and present some critical challenges to their use. We end with suggestions for further research to improve the scope, inclusivity, and technologies of participatory spatial tools for landscape governance.

## Integrated and Inclusive Landscape Governance

Whereas Noss (1983) coined the term ‘landscape approach’ to holistically address the preservation of regional diversity (Reed et al. 2020a), Görg (2007) was the first to frame this as landscape governance, soon followed by Beunen and Opdam (2011). Görg focused on multilevel decision-making as the outcome of interactions between ‘socially constructed spaces’ and the ‘biophysical conditions of place’. Without providing a clear-cut definition of landscape governance, he emphasized that it should take account of (i) social and cultural shaping of landscapes, (ii) a problem orientation that requires interdisciplinary cooperation, (iii) the plurality of landscape comprehensions and interests, (iv) a variety of human interferences with nature, resulting in a range from ‘quasi-natural’ protected areas to urbanized landscapes, and (v) a hybrid focus that goes beyond only the preservation of natural or cultural landscapes. In other words, ‘Cultural, aesthetic, economic and social dimensions are as much involved [in landscape governance] as ecological functioning or abiotic conditions’ (Görg 2007: 260–61). The various dimensions imply the need for a collaborative approach to accommodate the—often diverging—interests and values of multiple actors (Westerink et al. 2017). Drawing from Kooiman et al. (2005) and van Oosten et al. (2014), we define landscape governance as multisector, multi-actor, and multilevel interactions to solve societal problems and create societal opportunities at the landscape level (Ros-Tonen et al. 2014). More specifically, landscape governance is concerned with ‘the institutional arrangements, decision-making processes, policy instruments and underlying values in the system by which multiple actors pursue their interests in sustainable food production, biodiversity and ecosystem service conservation and livelihood security in multifunctional landscapes’ (Kozar et al. 2014: 2).

This conceptualization of landscape governance reflects three normative foundations. The first is the consensus- and solution-oriented interpretation of landscape governance: the ultimate objective is to negotiate attainable trade-offs between competing land uses (Sayer et al. 2013; Reed et al. 2016). Participatory spatial tools in this context enhance stakeholder engagement and collaboration and help clarify diverging claims and visions while giving voice to the less powerful.

The second underlying norm is to achieve sustainable, multifunctional landscapes that foster the conservation of biodiversity and ecosystem services, food and livelihood security, and climate resilience. While Görg (2007) argues that conserving natural or cultural areas is not an inherent normative foundation of the landscape governance concept and only one of the various possible outcomes, we observe that most authors associate the outcome of landscape governance with multifunctional landscapes that accommodate multiple interests. This is true of much of the literature on integrated landscape approaches (Minang et al. 2014; Freeman et al. 2015; Hart et al. 2015; García-Martín et al. 2016; Reed et al. 2020a) as well as other contributions to this special issue (e.g., Asubonteng et al. 2021; Djenontin et al.. 2021; Best et al. 2021; Shantiko et al. 2021).

The third foundation is the notion of inclusive landscape governance (Kusters et al. 2020). Inclusivity goes beyond the notion of stakeholder involvement as ‘invited participation’ (Cornwall 2002) or ‘tokenism’ (Somuah 2018). Inclusive development theory (Gupta et al. 2015a, b; Hickey et al. 2015) foregrounds marginalized peoples and reducing inequalities. This implies, among other things, (i) building on local and indigenous knowledge while making scientific knowledge available in the process, (ii) equal opportunity for participation, (iii) context specificity, i.e., alignment with local people’s realities and aspirations, (iv) targeted capacity building, (v) recognition of customary and traditional rights, and (vi) stimulating autonomy of the poorest (Gupta et al. 2015b; Ros-Tonen et al. 2019).

Due to the focus on multi-actor deliberations on trade-offs between competing land uses, landscape approaches can be considered forms of negotiated landscape governance. This entails that marginalized people have a voice in negotiating and shaping spaces. In practical governance terms, inclusivity requires the socio-spatial responsibility and entitlement to manage the holistic areas known as ‘landscapes’. Taking this further, some argue that while the concept of the holistic landscape is essential for understanding the dynamic interactions, the actual governance and management should focus more on spaces or ‘territories’ over which local communities or indigenous peoples have some form of designated ownership (Clay 2016; McCall 2016; Schroeder and González 2019; Windey and Van Hecken 2021). Hence, beyond advocating multi-actor and multisector collaboration, they emphasize the need for control and self-determination, particularly in indigenous lands. Participatory spatial tools are being deployed to achieve marginalized people’s inclusion in landscape governance.

## Participatory Spatial Tools

The use of spatial tools to engage people in rural development goes back to the ‘participation turn’ in the late 1970s and 1980s (Chambers 2006; Ellis and Biggs 2001) and has developed conceptually and technically since. This can be traced back to the following roots (c.f. McCall 2021):

1. Demand for people’s participation. This is a universal, progressive driver toward greater decentralization, accountability, popular democracy, and empowerment. Participation strengthens feelings and narratives of agency in the public space, i.e., for citizens to feel more included and engaged. It is a key component in the core categories of ‘good governance’. Post-normal science frames this as the ‘democratization of expertise’, and a reaction against long-running trends of ‘the scientization of politics and the politicization of expertise’ and against ‘the tendency towards assigning to experts a critical role in policy-making while marginalizing laypeople’ (Carrozza 2015: 109–110; see also Turnhout et al. 2010; Haklay 2013; Caquard 2014; Cavalier and Kennedy 2016). Within such a framing, citizens have more reason to want to be involved in landscape decision-making.
2. Indigenous/local land claims. The drive for deeper authentic participation has significantly reinforced the demands of indigenous and localized groups for official recognition of their territories and land and resource tenure rights. Participatory spatial tools have proven immensely productive in identifying and delineating indigenous and local land claims and in the translation into the formal mapping necessary for legitimizing and codifying these claims at the state level (McCall and Minang 2005; Sletto et al. 2020; Movik et al. 2021; Shantiko et al. 2021). In turn, the growing demand worldwide for regularizing indigenous land claims has further stimulated the development of effective participatory tools and modalities, particularly in the Global South (e.g., Lucas et al. 2018a, b).
3. The recognition that the (spatial) knowledge of local citizens has validity. This is the growing acceptance of the value and social-political significance accorded to the phenomenological and technical knowledge expressed by ‘ordinary people’. People’s local spatial knowledge relates to most elements of their lives, places, and livelihoods—their landscapes, territories, resource management, security, and conflicts. Civil society and decision-makers have learned to value citizen’s spatial information and knowledge as complementing conventional scientific knowledge. As a result, in many participatory spatial tools, local spatial knowledge may be prioritized over ‘codified’ knowledge (Warf and Sui 2010; Vogt et al. 2016; Young and Gilmore 2017; Aggrey et al. 2021; Somuah et al. 2021). This driver also appears in the Citizen Science principles of respect for local knowledge, committedness, and promoting people’s participation in scientific research (Haklay 2013; Robinson et al. 2018). This alternative ‘people’s knowledge’ is frequently critical of prevailing authorities and may disrupt social-political systems by contesting the sources and presentations of authoritative spatial information (Rambaldi 2005; Radil and Anderson 2019; McCall 2021). Thus, participatory processes in decision-making or policy formulation (for instance, the Intergovernmental Science-Policy Platform on Biodiversity and Ecosystem Services (IPBES) process on Indigenous and Local Knowledge) often contribute to conflicts between (hegemonic) official knowledge and people’s knowledge (Haklay 2013; Radil and Anderson 2019; Kyem 2021). Therefore it is crucial that these tools explicitly acknowledge the rights of the people as owners of their knowledge to safeguard their access, control, and autonomy (c.f. McCall 2016; MacKenzie et al. 2017; and the mandate of the World Intellectual Property Organization, WIPO 2020).
4. Advances in technological capacities. The final driver toward the increasing use of participatory spatial tools is the fast-growing easy access to technical capacities that enable quicker and broader public involvement through the internet and virtual communities and platforms. New technologies give unprecedented communication possibilities to mobilize citizens and activists and negotiate with institutional actors. These include affordable, accessible ‘WebGIS’ options, Geotagging tools, wiki-mapping with OpenStreetMap or Google Earth maps, spatial survey apps for smartphones and tablets, and open-source GIS software packages (e.g., QGIS) (Willemen et al. 2014; Voinov et al. 2016; Fagerholm et al. 2019; Kyem 2021).

## Discussion: Implications for a Future Research Agenda

Integrated approaches and using participatory spatial tools are not new, but their combination requires a new research agenda. We argue that further research is needed to expand the scope of application of the tools, improve inclusivity in the processes, and develop new technologies.

Regarding the scope of application, the challenge is to extend the use of participatory spatial tools to:

- Incorporate also the external drivers and agents of landscape change. The drivers of landscape degradation are mostly location-specific but also result from distant socioeconomic and environmental interactions, as made clear by Djenontin et al. (2021) and telecoupling literature (Liu et al. 2013; Eakin et al. 2014; McCord et al. 2018). Web-based applications have been developed to systematically map and analyze such interactions (e.g., McCord et al. 2018). Still, the challenge is to include both the local actors and the external agents of landscape change in the new applications.
- Continual monitoring. The potential of participatory spatial tools to monitor how the allocation of land and resource use and rights came into being (Shantiko et al. 2021) and how current trends shape (or interventions can change) transformations in the likely future (Asubonteng et al. 2021, Best et al. 2021) should be further developed. There is a need to go beyond ‘one-shot’ processes and turn ‘an experience of the future into a culture of the future’ (Shantiko et al. 2021) through continued monitoring of landscape change. This would also support the urgent need to develop more user-friendly participatory tools to monitor progress where integrated landscape approaches are being implemented (Sayer et al. 2013; Chervier et al. 2020).
- Combine with conventional spatial tools. Policymakers may not consider the results of participatory spatial tools as credible and acceptable knowledge, for instance, because they consider it an oversimplification of ‘reality’ (McCall 2016; Palomo et al. 2018; Best et al. 2021; Shantiko et al. 2021). In light of such conflicting epistemologies, several authors suggest the need for research to explore how the perceptions of past, present, and anticipated landscape dynamics drawn from participatory research compare with results of remote sensing analysis and modeling (Aggrey et al. 2021; Best et al. 2021; Somuah et al. 2021). In particular, further research on collaborative and participatory modeling is important (see, e.g., Voinov et al. 2016).

Inclusivity and representativity in the use of participatory spatial tools can be improved by:

- Extending participation to other actors and processes. Generally, participatory spatial tools are used in relatively closed, small, and homogenous groups in specific, focused projects. This close relationship between local participants and external collaborators may not permit sufficient ‘objective’ examination of how power imbalances influence their effectivity and inclusivity. By broadening the scope of actors, including the ‘bad guys’, it is possible to expand the knowledge inputs, be more representative (democratic), and reduce ‘parochialism’ or the ‘bubble effect’ (McCall 2021). Moreover, broadening the application, especially to local administrative authorities, allows an exploration of how participatory spatial tools can be integrated into formal planning processes (Do et al. 2021; Best et al. 2021).
- Improving the security and safety of the internal information networks of local and indigenous peoples and civil society organizations so that outsiders cannot hack their internal conversations on landscape governance or culturally sensitive information (Eshbaugh 2008; Barlindhaug and Corbett 2014). Cyber security per se (e.g., Brown and Nicholas 2012) has not yet received as much attention as privacy and confidentiality issues in general in the literature on spatial tools for landscape governance. Information protection plays a role in the paper by Aggrey et al. (2021), who show that participants in community mapping of (illegal) mining activities in Ghana prefer to withhold the names of their communities from the maps to avoid repercussions. In a similar vein, participants in the study by Somuah et al. (2021) decided not to map sacred groves and species of cultural and spiritual importance to prevent their disturbance by outsiders. These examples show that ‘inclusivity’ does not necessarily imply ‘unrestricted access and openness to all’.
- Strengthening inclusivity by making tools more user-friendly for older, disabled, and low-literate people uncomfortable with these technologies. This includes ensuring better access options for marginalized people (e.g., poor people, women, and minority groups) in terms of language, internet coverage, and affordable devices (c.f., Best et al. 2021) and promoting broad access to and documentation of data for inclusive use options.
- Paying priority attention in research and training to ethical issues, especially regarding who determines what is mapped, and by whom, and for what uses in the short and long terms, who benefits, and who eventually owns the map (Rambaldi et al. 2006; Verplanke et al. 2016; Aggrey et al. 2021; Somuah et al. 2021).

Potentials for upcoming and new technologies requires examination of:

- Community-managed and -controlled unmanned aerial vehicles (UAVs), which have a proven potential as a spatial tool for community landscape governance (e.g., Vargas-Ramírez and Paneque-Galvez 2019).
- Improved basic field technologies, i.e., tougher tablets, robust batteries for more durability in the field, and devices allowing to read a tablet or smartphone in bright sunshine (see also Palomo et al. 2018).
- Explore new materials that allow easy adjustment of the surface image of 3D models. This can be deftly performed with digital 3D models. However, most large physical 3D models, including those used in participatory 3D modeling, tend to remain static. Once created, they can become immutable artifacts because technical constraints and psychological barriers may hinder overlaying or repainting them (Chassin et al. 2019). Creating adjustable, malleable, physical 3D models requires exploring new materials that allow easy adjustment of the surface image.

## Conclusions

A growing body of literature (including in this special issue) vindicates the use of participatory spatial tools to uncover and visualize stakeholder perspectives of landscape dynamics and associated conflicts as a basis to negotiate solutions. This literature is indicative of the increasing endorsement of participatory spatial tools in place-based landscape governance. From an inclusive development perspective, participatory spatial tools may enhance the empowerment of marginalized people and give them a voice in landscape governance by making their interests and claims spatially explicit. However, spatial tools may also obscure the complexity of landscapes and associated power dynamics, and some limitations challenge their inclusivity. More research is needed to explore how these challenges can be met and more inclusive landscape governance be achieved by extending the scope, representativity, and technology of participatory geospatial tools.

## Footnotes

1 The actual implementation of integrated landscape approaches is still in its infancy, but several initiatives have been documented (Milder et al. 2014; Hart et al. 2015; García-Martín et al. 2016; Zanzanaini et al. 2017) and efforts toward their further operationalization are underway (Freeman et al. 2015; Reed et al. 2020b).

2 Some of these articles are referred to in this paper (Aggrey et al. 2021; Asubonteng et al. 2021; Best et al. 2021; Djenontin et al. 2021; Do et al. 2021; Shantiko et al. 2021; Somuah et al. 2021).
